# Establishing a reliable protoplast system for grapevine: isolation, transformation, and callus induction

**DOI:** 10.1007/s00709-025-02069-7

**Published:** 2025-04-25

**Authors:** Gulsen Kolasinliler, Cengiz Akkale, Hilal Betul Kaya

**Affiliations:** 1 Department of Bioengineering, Faculty of Engineering, Manisa Celal Bayar University, Manisa, Türkiye; 2 Izmir Biomedicine and Genome Center, İzmir, Türkiye

**Keywords:** Protoplasts, Mesophyll cell, Grapevine, Protoplast isolation, Transient transformation

## Abstract

Protoplasts are single cells enclosed by the plasma membrane after cell wall removal. They are widely used in various biotechnological applications, including gene functional analysis, verification of genome editing reagents, and plant regeneration. Recent advances in genome editing have enabled the production of non-chimeric and transgene-free genome-edited plants using protoplasts. This process involves protoplast isolation, transformation, and regeneration, requiring advanced technical skills. Challenges in isolation and regeneration have limited their use in genome editing. In grapevines, however, very few studies have reported the use of protoplasts isolated from leaves. Efficient isolation and transformation protocols for Chardonnay remain lacking and require cultivar-specific optimization. In this study, we established a reliable and efficient protoplast isolation and transformation system by optimizing conditions for protoplast isolation and PEG-mediated transformation in Chardonnay cultivar. The yield of viable protoplasts was approximately 75 × 10^6^ per gram of leaf material, with a viability of 91%. A transformation efficiency of 87% was achieved under the optimized conditions. To evaluate the regeneration ability of mesophyll protoplast, transformed and untransformed protoplasts were cultured on solid and liquid MS media supplemented with 2 mg/L 2,4-D and 0.5 mg/L BA to facilitate microcalli formation. Microcalli formed on the feeder layer and developed into calli when transferred to liquid MS culture with 2 mg/L 2,4-D and 0.5 mg/L BA. However, the calli were unable to regenerate into roots or shoots. These findings provide a foundation for further optimization of protoplast-based regeneration systems in grapevines, with the potential to enhance genome editing applications in this species.

**Supplementary Information:**

The online version contains supplementary material available at 10.1007/s00709-025-02069-7.

## Introduction

Viticulture is one of the highest-value agricultural sectors directly affected by climate change (Venios et al. 2020; Kaya et al. 2023). Over the last few decades, viticulture has faced significant threats and challenges due to factors such as water and soil conditions, temperature, radiation, atmospheric conditions, pathogens, and greenhouse gas concentration (Moretti et al. 2010; Pastore, Frioni, Diago 2022; Tosato, Van Volkenburg, Vasseur 2023). Although conventional breeding has been the primary method for improving grapevines, developing a new cultivar is time-consuming and labor-intensive (Campos et al. 2021; Zhang et al. 2018). Additionally, the traditional crossing is often avoided in wine grapes due to the desire to preserve clonal genetic integrity (Giacomelli et al. 2023). Genome editing offers a powerful alternative to conventional breeding for trait improvement (Najafi et al. 2023; Ceasar and Kavas 2024; Ricroch et al. 2024). This technology plays a crucial role in enhancing yield, quality, disease resistance, and stress tolerance in various plants (Cardi et al. 2023), including grapevine (Ren et al. 2020, 2023; Giacomelli et al. 2023; Butiuc-Keul and Coste 2023). Genome editing relies on inserting editing components into plant cells and regenerating edited plants (Kaya et al. 2021). During this process, tissue culture is the most challenging step, as it is time-consuming, labor-intensive, and genotype-dependent (Son and Park 2022; Wada et al. 2020). Protoplasts, which are plant cells devoid of cell walls, provide an alternative and rapid strategy for assessing multiple genome editing reagents rapidly, such as sgRNAs, Cas proteins, and promoters (Nadakuduti et al. 2019; Yue et al. 2021). Instead of transfecting CRISPR reagents directly to the plants, researchers increasingly rely on protoplasts as a versatile tool to evaluate the effectiveness and precision of gene editing. This approach enables the swift assessment of CRISPR reagents’ performance, providing valuable insights before advancing to complete plant transformations (Jiang, Bush, Sheen 2021). Protoplasts also offer a convenient platform for evaluating protein localization, DNA–protein interactions, protein–protein interactions, and functional characterization of genes (Gilliard et al. 2021; Jung et al. 2015; Yue et al. 2021). Additionally, to enable DNA-free genome editing, protoplasts provide an alternative strategy with their ability to regenerate full plants (Scintilla et al. 2022). Plant protoplasts have been successfully isolated and transformed from various crops, including *Arabidopsis*, tobacco, rice, wheat, maize, lettuce, carrot, and tomato (Jiang, Bush, Sheen 2021; Lin et al. 2018a, b).

Although protoplast isolation and transformation have been implemented in woody plant crops such as citrus, apricots, peaches, tea plants, and grapevines, protoplast isolation remains more challenging (Kuzminsky et al. 2016; Nerva et al. 2023). The efficiency of protoplast isolation varies between different plant types and among different cultivars of the same plant. Different explant types from the same cultivars can affect protoplast yield (Ren et al. 2021). Protoplast transformation is a rapid and efficient method compared to *Agrobacterium*-mediated plant transformation for testing purposes, particularly for transient expression studies (Gou et al. 2020; Lin, Chen, Fang 2018; Akkale 2025). An efficient protoplast transformation is paramount, given the focus on obtaining transgene-free-genome-edited plants and using protoplasts as a testing platform. In protoplast isolation, obtaining healthy and large numbers of protoplasts, achieving high transformation efficiency, and preserving the health and viability of transformed protoplasts are critical.

Various types of explants, such as berries (Fontes et al. 2010), leaves (Mliki et al. 2003; Zhao et al. 2016), and callus tissues (Malnoy et al. 2016), have been used in protoplast isolation and transformation from grapevine. Although research on grapevine-derived protoplasts began in 1985, isolating, transforming, and regenerating whole plants from protoplasts remains challenging (Bertini et al. 2019; Yue et al. 2021).

While protoplasts are useful for studying cellular functions, regenerating whole plants is crucial for understanding how genes impact plant development or physiology. This represents a significant hurdle in many plant species, including grapevines, which are particularly difficult to regenerate (Bertini et al. 2019). Grapevine regeneration and the isolation of regenerative protoplasts present significant challenges (Derman and Vivier 2022). Ongoing protocol optimizations address these challenges by focusing on different grapevine varieties to achieve viable and regenerative protoplasts.

This study presents a simplified and highly efficient protoplast isolation and transformation method from grapevine (*Vitis vinifera* L.cv. Chardonnay) leaves. Several critical factors affecting protoplast isolation, including leaf age, leaf amount, cutting style of leaves, mannitol implementation as a pre-treatment, cell wall digestion time, filtration with different pore sizes, and separation by sucrose gradient and ice incubation were investigated. The transformation protocol was also optimized based on the protoplast number, PEG incubation time, and incubation duration after transformation. As part of the study, callus induction from transformed and untransformed protoplasts was compared using solid and liquid MS (Murashige and Skoog) media supplemented with 2 mg/L 2,4-D (2,4-Dichlorophenoxyacetic acid) and 0.5 mg/L BA (6-Benzylaminopurine). These findings provide a robust protoplast isolation and transformation protocol to advance genome editing studies in grapevine, particularly in the studies for gene functional analysis, construct validation, and transgene-free genome editing.

## Materials and methods

### Plant material

Chardonnay cuttings were grown in a growth chamber with temperatures of 17 °C at night and 25 °C during the day. Relative humidity was maintained between 60 and 80% during a photoperiod of 16 h. Freshly cut young leaves were used as explants for each protoplast isolation experiment.

### Plasmid preparation

The pMOD_C3001 vector (Čermák et al. 2017), containing CaMV 35S promoter and GFP gene (with a *Bae*I site for GT donor cloning), was used for transformation. *E. coli* (DH5α) transformed with pMOD_C3001 plasmid were grown in 100 mL of Luria–Bertani (LB) liquid medium supplemented with 100 µg mL^−1^ carbenicillin in a 1 L baffled flask with shaking at 250 rpm at 37 °C for 16 h to reach an OD of 0.4. Plasmid DNA was extracted following the manufacturer’s recommended protocol using the Macherey–Nagel Xtra Midi Prep Kit (GmbH&Co. KG, Germany). Ten micrograms of plasmid DNA was used for each transformation.

### Protoplast isolation

Protoplast isolation from grapevine leaves was performed following the methods described in previous reports (Shan et al. 2014; Yoo, Cho, Sheen 2007; Zhao et al. 2016). A series of experiments were conducted, optimizing one factor at a time to maximize the yield of healthy protoplasts. The parameters implemented and compared are detailed in Table 1 below.

**Table 1:** Optimization factors for protoplast isolation efficiency

Factor	Description
Factor 1—Leaf age and amount	20 mg young leaves
	40 mg young leaves
	20 mg mature leaves
	40 mg mature leaves
Factor 2—Cleaning/sterilization of leaves	Surface sterilization of leaves by submerging in 5.25% sodium hypochlorite for 1 min followed by 70% ethanol for 2 min, and lastly rinsing 4 times with distilled water
	Addition of antibiotics in enzymatic digestion solution
	Washing with sterile distilled water only
Factor 3—Cutting method	Random cutting: All parts of washed leaves were randomly cut into approximately 0.1–0.5 mm pieces with a razor blade
	Tape-sandwich: Supporting the top side of the leaf with tape while using another tape to remove the lower epidermal layer
	Strip-cutting: Leaves were gently shredded into 0.5–1.0 mm strips on filter paper by a razor blade, and the petiole was discarded
Factor 4—Pre-treatment	Incubation with mannitol (0.6 M) before enzymatic digestion
	No pre-treatment
Factor 5—Incubation time with enzyme solution in the dark	4 h
	8 h
	16 h
	20 h
Factor 6—Separation of protoplasts from enzymatic solution	Filter with a pore size of 40 µm (Corning® 40 µm)
	Filter with a pore size of 70 µm (Corning® 70 µm)
Factor 7—Separation of protoplasts after washing with W5	Pipetting supernatant after ice incubation
	Sucrose gradient (from 10 to 30%)
	Centrifuge at 150 × g using a swinging rotor

Additionally, certain protocol steps remained consistent throughout the experiments. Cell wall digestion was carried out with the enzyme solution (1.5% cellulase R10 (Yakult, Tokyo, Japan), 0.75% macerozyme R10 (Yakult, Tokyo, Japan), 0.6 M mannitol, 10 mM CaCl_2_, 0.1% BSA and 10 mM MES, pH 5.7) which was found to be the most effective based on the literature (Shan et al. 2014). The cut leaves (see Factors 1, 2, 3, 4) were transferred to a Petri dish containing 1 g of leaf material per 50 mL of enzyme solution and placed in a vacuum chamber for vacuum infiltration at ~ 400–500 mmHg for 30 min in the dark, with vacuum cycles alternating every 10 min. The vacuum was then released, and the strips were incubated (see Factor 5) with enzyme solution in the dark with gentle shaking (50 rpm) on a shaker at room temperature. Following the digestion process, an equivalent amount of W5 buffer (containing 2 mM MES (pH 5.7), 154 mM NaCl, 5 mM glucose, 125 mM CaCl2, and 5 mM KCl) was added and mixed gently for 1 min to stop the digestion and release the protoplasts. Protoplasts were filtered through a nylon mesh (see Factor 6), pre-wetted with 1 mL of W5 buffer, and centrifuged at 150 × g with gentle acceleration and deceleration for 5 min at room temperature (RT) in a swinging bucket rotor. The supernatant was removed, and 20 mL of W5 buffer was added to the pellet to wash the protoplasts and resuspend them. The W5 buffer was then removed (see Factor 7), and the protoplasts were resuspended in 2 mL of MMG (4 mM MES (pH 5.7), 0.4 M mannitol, 100 mM CaCl_2_ and 15 mM MgCl2). Each experiment included three independent replicates.

### Protoplast yield and viability

The protoplasts were counted and photographed using an Olympus CKX53 microscope (Olympus, Tokyo, Japan) with a hemocytometer. Protoplast yield was calculated as the number of protoplasts per gram of fresh weight (FW) leaf tissue used for isolation. The viability of protoplasts was assessed by fluorescein diacetate (FDA) staining. FDA, dissolved in DMSO (5 mg/mL), was added to the protoplast solution to a final concentration of 0.05% and incubated in the dark for 2 min. FDA-stained protoplasts were observed using an Olympus IX53 inverted fluorescence microscope (Olympus, Tokyo, Japan). Viable cells fluoresced green after staining, while dead cells and cellular debris remained dark. The viability of protoplasts was calculated as follows:

$$\text{Viability }\left(\text{\%}\right)=\left(\frac{\text{Total fluorescent protoplast number}}{\text{Total protoplast number}}\right)\times 100$$

### PEG-mediated transformation

Transformation of grapevine protoplasts was performed using a polyethylene glycol (PEG)–Ca^2+^ transformation method with some modifications (Shan et al. 2014; Yoo, Cho, Sheen 2007; Zhao et al. 2016). The procedure was optimized according to the key steps outlined in Table 2 below to increase transformation efficiency.

**Table 2:** Optimization factors for protoplast transformation efficiency

Factor	Description
Factor A—Protoplast number	2.5 × 10^5^
	5 × 10^5^
	1 × 10^6^
	2 × 10^6^
Factor B—PEG incubation time	2 min
	5 min
	10 min
	15 min
Factor C—Incubation buffer after transformation	W5
	MMG
Factor D—Incubation time after transformation	16 h
	48 h

For each transformation, 10 µg of plasmid DNA was gently mixed with 200 µL MMG-protoplast mixture (see Factor A) in 2 mL round-bottomed centrifuge tubes. An equal volume of freshly prepared and filter-sterilized PEG solution (40% (w/v) PEG- 4000 in MMG buffer) was added and mixed gently. The protoplast-PEG solution mixture was incubated at room temperature in the dark (see Factor B). After transformation, 900 µL of W5 buffer was added to stop the transformation. Protoplasts were collected by centrifuging at 100 × g for 5 min at room temperature. After removing the supernatant by pouring, the protoplasts were resuspended (see Factor C), transferred to 6-well plates, and incubated in the dark (see Factor D) for further analysis. Each factor in the transformation experiment was tested in three independent replicates.

### Fluorescence microscopy

Protoplasts were observed using an Olympus IX53 inverted fluorescence microscope (Olympus, Tokyo, Japan) with GFP excited at 488 nm. The transformation efficiency was calculated as follows:

$$\text{Transformation efficiency }\left(\%\right)=\left(\frac{\text{Transformed protoplast number}}{\text{Total protoplast number}}\right)\times 100$$

### Confirmation of transformation with PCR

PCR analysis was performed to confirm the presence of GFP in the transformed protoplasts. Genomic DNA was extracted from the transformed protoplasts using the method described by (Oncu-Oner et al. 2023). PCR primers were designed (GFP forward: ctgatcatatgaagcggcacgact, GFP reverse: gccatgtgtaatcccagcagctg), and the Q5® High-Fidelity 2X Master Mix (NEB, MA, USA) was used under the following conditions: initial denaturation at 94 °C for 30 s, followed by 30 cycles of denaturation at 94 °C for 30 s, annealing at 60 °C for 30 s, and extension at 68 °C for 80 s, with a final extension at 68 °C for 5 min. DNA from wild-type Chardonnay was used as the negative control. PCR products were visualized by electrophoresis on a 1% (w/v) agarose gel.

### Protoplast culture for callus induction

**Fig. 1:**
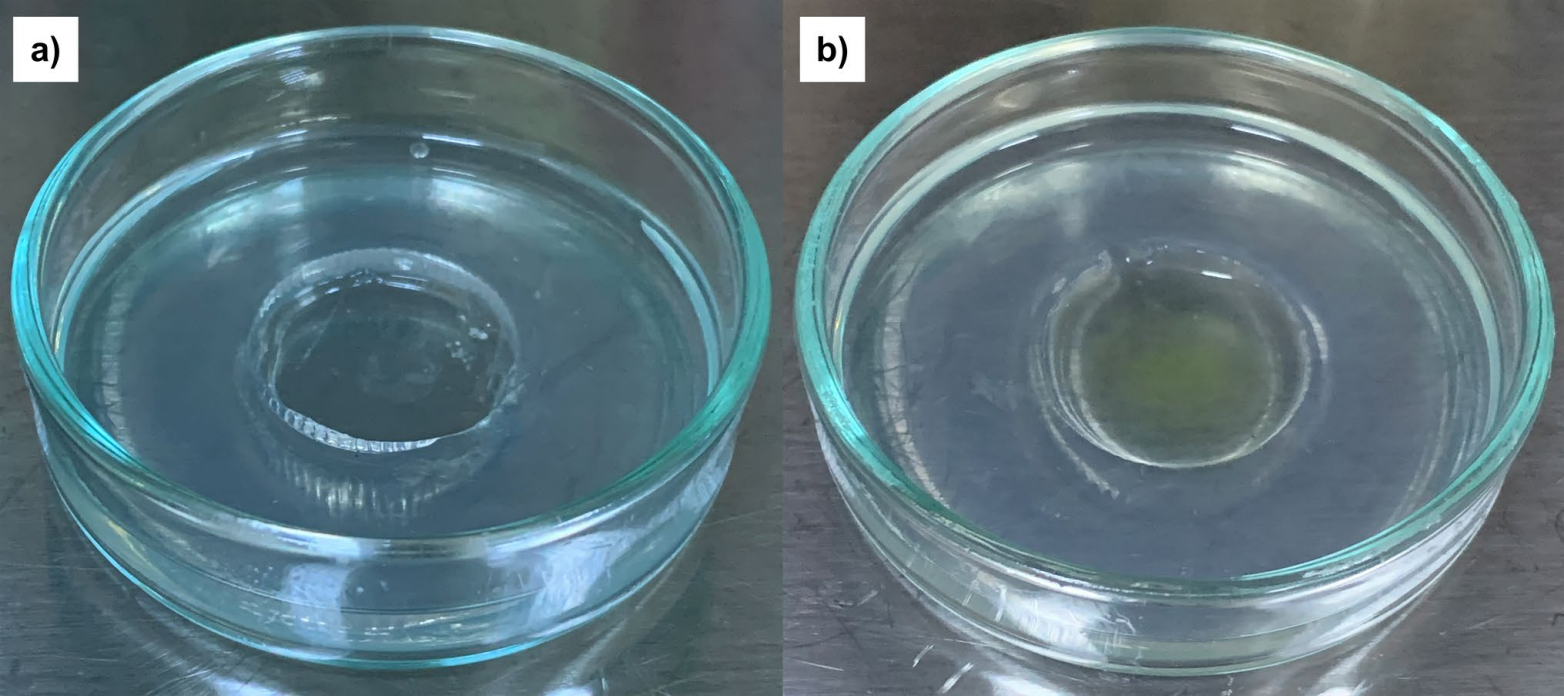
Formation of agar pool and addition of protoplast-liquid culture medium mixture. **a** Before addition of protoplasts. **b** After addition of protoplasts

One million protoplasts in 100 µL of MMG were inoculated into liquid or agar-pool media. The MS medium (500 mg/mL casein, 0.3 M mannitol, 2% sucrose, 1% glucose, pH 5.8) was used for both culture systems. In the liquid culture system, protoplasts were incubated in 4 mL of liquid medium supplemented with 1 µL/mL carbenicillin and the required hormones (2 mg/L 2,4-D and 0.5 mg/L BA). For the agar-pool culture, 0.85% (w/v) agar was used to solidify the MS medium. The agar-pool culture was prepared as described by Hu et al. (Hu, Yin, He 2015), with modifications. This system poured 4 mL of molten agar medium with 1 µL/mL carbenicillin into Petri dishes. Once solidified, the inverted cap of a 15 mL Falcon tube was placed on the agar surface, and an additional 4 mL of molten agar medium with carbenicillin was added around the cap. After solidification, sterile hot water was introduced into the cap to partially melt the surrounding agar for easy removal, forming the agar-pool. A 500 µL mixture of 1 × 10⁶ protoplasts in liquid medium, with carbenicillin and hormones (2 mg/L 2,4-D and 0.5 mg/L BA), was then added to the agar pool (Fig. 1). Protoplasts were incubated in both systems under dark conditions at room temperature for 30 days, followed by biweekly subculturing with the same media for 2 months. After 2 months, the cultures were transferred to 100 mL Erlenmeyer flasks containing liquid medium with the same hormone combination for large-scale cultivation. After 15 days, filtration separated the microcalli from the medium, and the fresh medium was added to the Erlenmeyer flasks. After another 30 days, the microcalli were filtered through a nylon mesh and transferred to the MS agar medium with various hormone combinations, supplemented with solid feeder layers (1 mg/mL BA, 0.25 mg/mL NAA (naphthaleneacetic acid), and 1 mg/mL TDZ (thidiazuron), 0.25 mg/mL NAA) to promote the formation of embryogenic calli. All experiments were performed in triplicates. The calli, which did not grow in the final stage on the agar medium, were transferred to MS and Lloyd (Lloyd and McCown’s Woody Plant Medium) liquid media (Lloyd and McCown 1980) with the same hormone combinations (1 mg/mL BA, 0.25 mg/mL NAA, and 1 mg/mL TDZ, 0.25 mg/mL NAA).

## Results and discussion

### A high-efficiency method of protoplast isolation from Chardonnay leaves

**Fig. 2:**
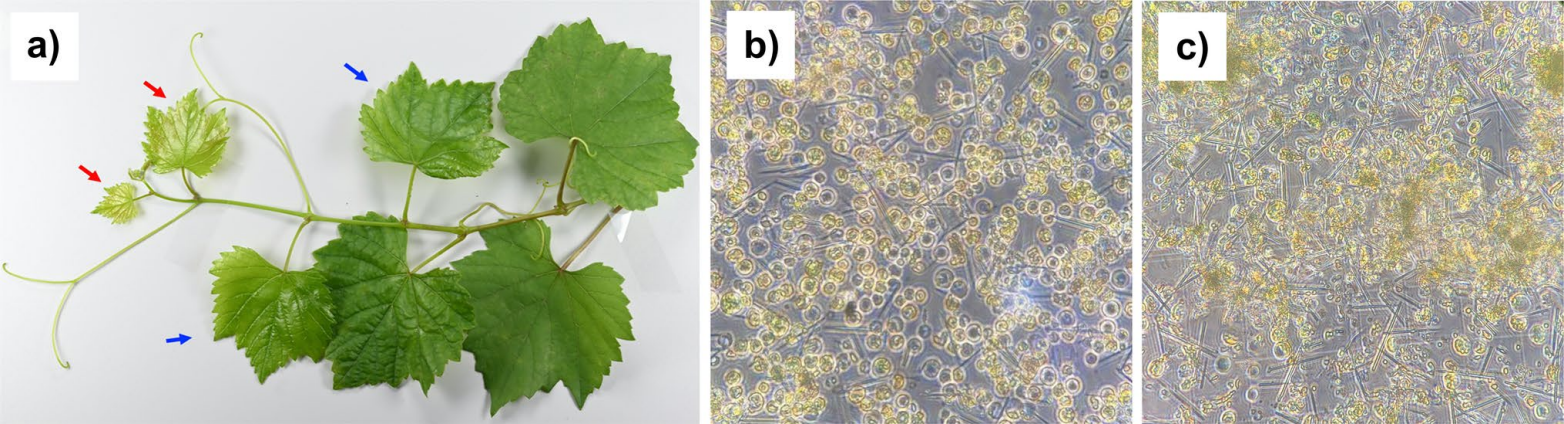
Effects of leaf age and amount of grapevine leaves on protoplast isolation. Young (1 st and 2nd apical leaves, indicated with red arrows) and old (3rd and 4 th leaves, indicated with blue arrows) leaves (**a**). Protoplasts obtained with 20 mg (**b**) and 40 mg (**c**) of young leaves

A series of factors were evaluated in the study to optimize a high-efficiency protoplast isolation protocol for grapevines. Various optimization steps were systematically implemented for a high yield of viable protoplasts. Since selecting the proper source and amount of leaf material is critical, leaf age and amount were considered the first optimization parameters. Leaves at different growth stages (young leaves: first to 2nd; old leaves: 3rd to 4 th) were utilized, as illustrated in Fig. 2a. The yield of protoplasts from both young and old leaves, depicted in Fig. 2b and c, was compared at 20 mg and 40 mg leaf per mL of extraction buffer. The results indicated that both the age and amount of leaf tissue significantly influenced protoplast release. When 20 mg of young leaves per mL of extraction buffer was utilized, the isolated protoplast yield peaked at 75 × 10^6^ protoplasts/g leaf. Conversely, protoplast yield from old leaves remained low regardless leaf amount, accompanied by substantial debris compared to young leaves. Protoplasts obtained using 20 mg and 40 mg of young leaves per 1 mL of extraction buffer are shown in Fig. 2b and c, respectively. These microscopy images show that protoplasts obtained with 20 mg of young leaves were clear of debris and had overall better spherical morphology (Fig. 2b). However, increasing the amount of leaves to 40 mg per 1 mL of extraction buffer deteriorated the digestion quality, resulting in fewer protoplasts and more debris.

Grapevine protoplasts can be isolated from various tissue and organ types, including leaves (Zhao et al. 2016), calli (Scintilla et al. 2022; Tricoli and Debernardi 2024), stems (Alleweldt 1988), berries (Fontes et al. 2010), and roots (Alleweldt 1988). These different tissue sources exhibit distinct characteristics and necessitate varied culture conditions (Fontes et al. 2010; Wang et al. 2015; Zhao et al. 2016). Existing protoplast protocols tailored for grapevine callus, stem, and berry tissues present limitations such as low yield and significant time investment (Fontes et al. 2010; Zhao et al. 2016). These constraints hinder their efficacy in facilitating genome editing studies (Li et al. 2021). The mesophyll tissues in leaves represent a highly convenient source of numerous uniform cells for isolating protoplasts (Yoo, Cho, Sheen 2007; Li et al. 2021). The age of the tissue also plays a critical role in determining both the yield and viability of protoplasts, especially in woody plants (Wakita et al. 1996; Zhao et al. 2016). Zhao et al. (2016) emphasized the importance of using young leaves for isolating protoplasts from *V. vinifera* cv. Rizamat and the wild Chinese grapevine. They reported that the yield of protoplasts decreases when isolated from older leaf tissue in grapevine (Zhao et al. 2016). In alignment with our results, it is worth noting that previous studies have also observed the challenge of obtaining ample amount of viable protoplasts from mature leaves across diverse plant species, including orchids, maize, and rice (Ren et al. 2021). Additionally, reports indicate that both the physiological conditions and the age of the leaves strongly influence the quality of isolated protoplasts (Jiang, Bush, Sheen 2021).

The effect of the amount of tissue for protoplast isolation was also investigated in previous studies. Zhao et al. (2016) reported that they obtained the highest yield (approximately 3 × 10^4^ protoplasts) when they used 20 mg of tissue per mL of extraction solution, which represented the highest leaf tissue to extraction buffer ratio implemented in their study. However, our study obtained a higher yield (7.5 × 10^6^ protoplasts) from 20 mg of tissue per mL of extraction solution. They also mentioned the importance of the leaf tissue-to-extraction buffer ratio for better breakdown. In our study, the cell wall was not effectively broken down when the leaf amount exceeded 20 mg per mL of extraction buffer.

In this study, we examined the effects of surface sterilization of grapevine leaves on protoplast isolation. We observed that sterilization with Clorox and ethanol decreased protoplast yield by damaging the leaf tissue. Additionally, adding antibiotics to the enzyme solution did not affect contamination or protoplast yield. Notably, when leaves were washed only with sterile distilled water before protoplast isolation, no contamination was observed if the experimental procedures maintained the aseptic technique. At the same time, surface sterilization of leaf samples has been implemented in previous protoplast isolation studies in grapevine (Wright 1985); the effects of this procedure were not addressed in this study. Other reports on mesophyll protoplast isolation did not mention any surface sterilization procedure for grapevine leaves (Zhao et al. 2016; Malnoy et al. 2016).

**Fig. 3:**
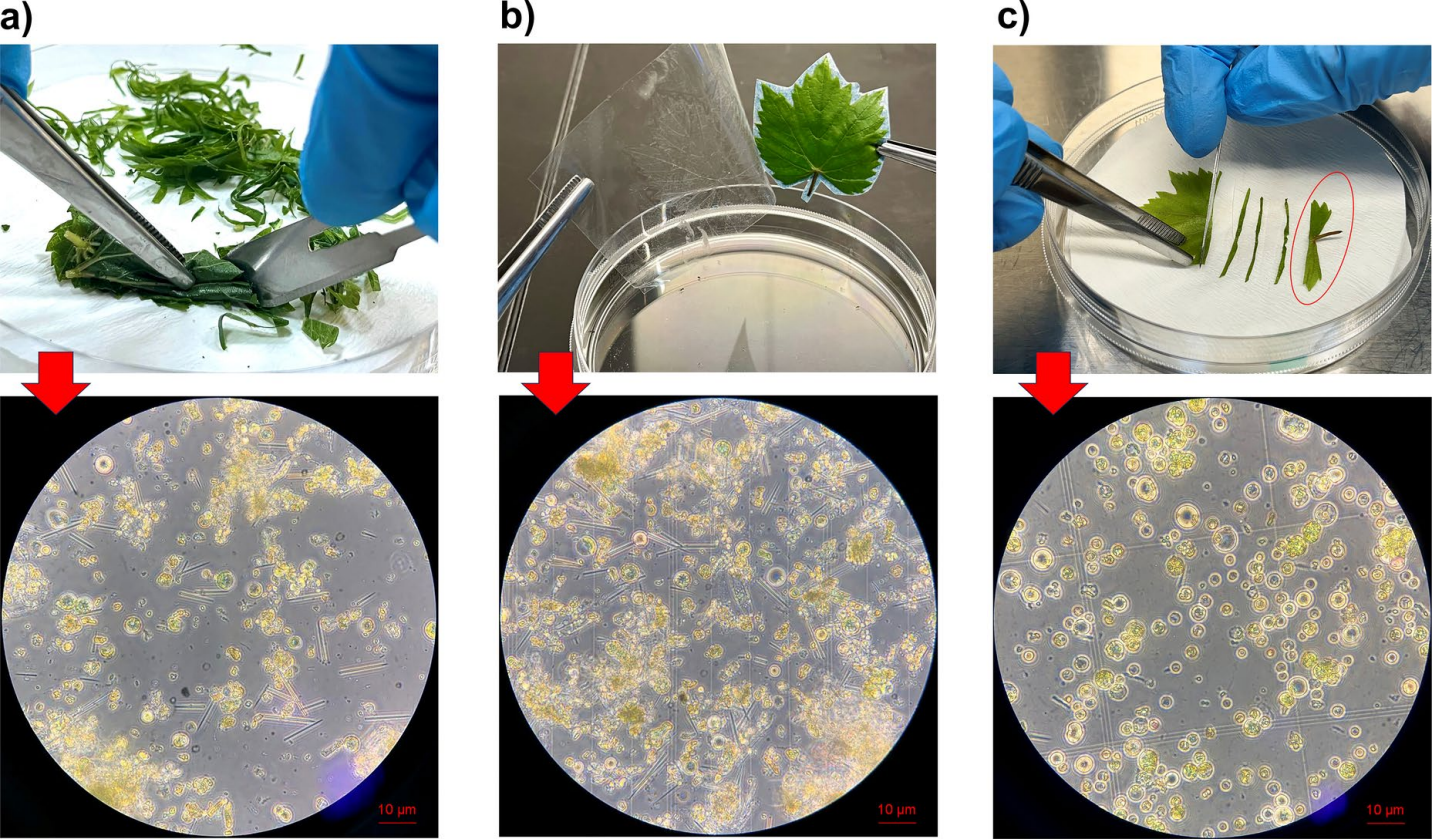
Leaf cutting methods employed in the study, alongside images of the resulting protoplasts observed under a bright-field microscope. **a** Random cutting. **b** Tape-sandwich. **c** Strip cutting (petiole is highlighted with red ellipse)

To determine the most efficient cutting method for protoplast isolation, we evaluated three different cutting methods in this study (Fig. 3). Our findings revealed that harsh, randomly applied bulk-cutting methods resulted in tissue damage and failed to yield healthy protoplasts (Fig. 3a). We also obtained undigested tissues, such as clumps and damaged protoplasts, when we implemented this technique. Additionally, we implemented the tape-sandwich method for the first time in grapevine leaves (Fig. 3b), which presented challenges compared to established methods used in other plant species such as *Arabidopsis* and *Artemisia japonica* (Wu et al. 2009; Deng et al. 2023). Furthermore, there are drawbacks associated with this method: the experimental procedure involving tape attachment is impractical, and there is a high risk of contamination. Also, similar to our study, the tape-sandwich method was the least efficient in potato protoplast isolation (Moon et al. 2021). In contrast to these challenges, a gentler cutting approach successfully obtained a high yield of protoplasts, consistent with previous findings in *Arabidopsis* (Yoo, Cho, Sheen 2007). Here, the leaves were gently sliced into 0.5–1.0 mm strips on the filter paper by razor blade after removing petiole part, as shown in Fig. 3c. The significance of the cutting method has been examined in other studies, highlighting the importance of gentle cutting techniques to obtain a high number of protoplasts (Wu et al. 2009; Moon et al. 2021; Deng et al. 2023). It is also important to avoid exposure to dry conditions within the laminar flow cabinet during cutting, and cuttings should be immediately transferred to the enzyme solution. In this study, needle-like structures were also observed during protoplast isolation, with a higher quantity present in the random-cutting method (Fig. 3a) and tape-sandwich method (Fig. 3b) compared to the strip-cutting method (Fig. 3c). These structures are likely to be raphides, tiny, needle-shaped calcium oxalate crystals that are commonly found in various plant species, including grapevine (Jáuregui-Zúñiga et al. 2003; DeBOLT et al. 2004).

**Fig. 4:**
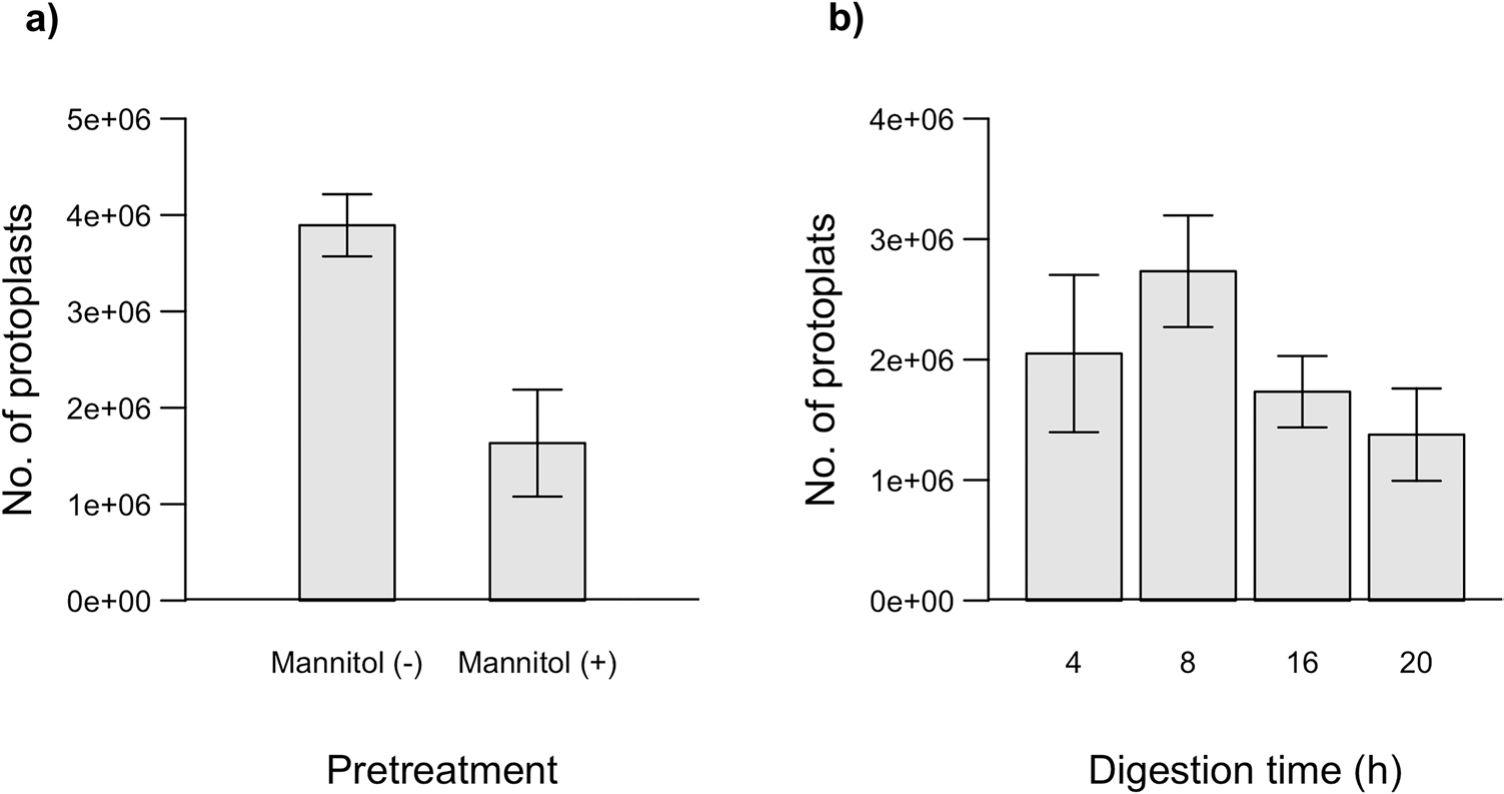
Effects of the mannitol addition (**a**) and digestion time (**b**) on the protoplast number

In the study, mannitol implementation as a pre-treatment was investigated as a potential optimization parameter. Previous protocols (Li et al. 2018; Sun et al. 2019; Deng et al. 2023) have suggested pretreating leaf samples of *Phalaenopsis*, *Brassica*, and *Artemisia japonic*a with 0.4–0.6 M mannitol to facilitate the efficient penetration of extraction buffer through the plant cell wall and membrane pores (Reed and Bargmann 2021; Li et al. 2022; Chen et al. 2023). However, despite employing a 0.6 M mannitol-mediated incubation for Chardonnay leaves, this pre-treatment step resulted in a 75% reduction in the number of protoplasts isolated (Fig. 4a). Previous reports on grape protoplast isolation have also refrained from implementing mannitol as a pre-treatment for leaves and callus explants (Zhao et al. 2016; Scintilla et al. 2022).

**Fig. 5:**
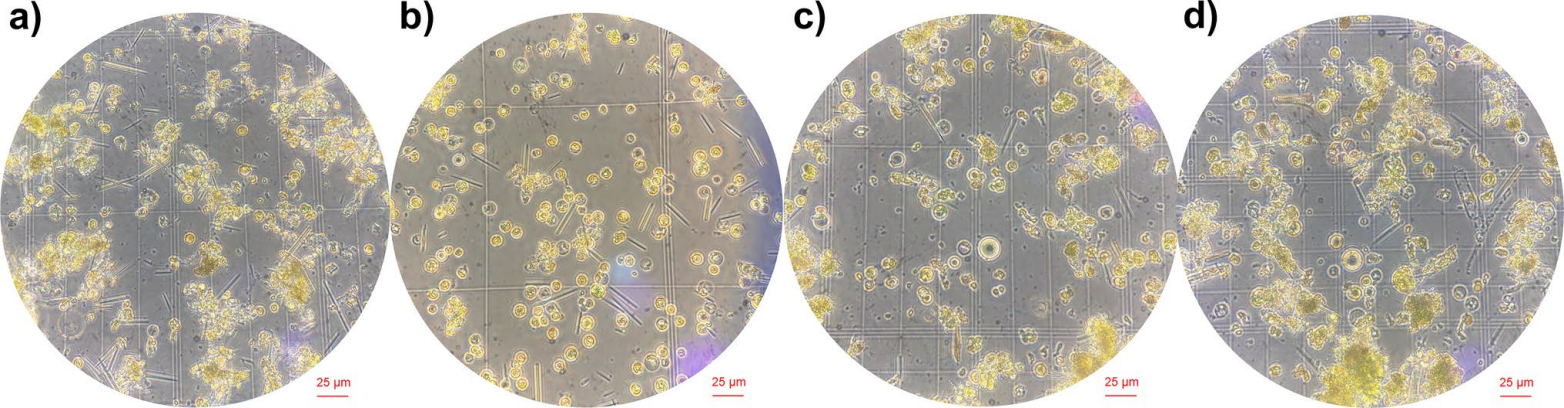
Protoplasts under inverted bright field microscope obtained by 4 (**a**), 8 (**b**), 16 (**c**), and 20 (**d**) hours of digestion incubation time

To investigate the influence of incubation times with enzyme solution and assess whether extended incubation periods yield any adverse effects, we evaluated four incubation times (4 h, 8 h, 16 h, 20 h). Our study revealed that an 8-h digestion time was optimal for leaf samples of Chardonnay. The number of protoplasts corresponding to each digestion time is shown in Fig. 4b. Notably, the number of protoplasts continued to increase until 8 h of digestion; however, prolonged incubation beyond this point resulted in a decline in protoplast yield. Protoplasts obtained from different incubation times were examined under an inverted bright-field microscope, as illustrated in Fig. 5. Analysis of the images revealed heterogeneous digestion of the plant cell wall in the 4 h of incubation. However, prolonged incubation periods of 16 and 20 h resulted in protoplasts rupturing and losing their characteristic spherical morphology. It has been noted that the optimal duration for cell wall digestion varies depending on several factors, such as the source material, research goals, intended outcomes, and the types and concentrations of enzymes used in isolating protoplasts (Yoo, Cho, Sheen 2007).

The initial investigation into protoplast isolation from grapevine leaf mesophyll tissues revealed that the optimal incubation period for achieving the highest yield of viable protoplasts ranged from 2 to 3 h, with a standard practice of two and a half hours (Barbier and Bessis 1990). Malnoy et al. (2016) utilized incubation durations of 4 h and overnight for protoplast isolation from Chardonnay, targeting embryogenic calli and leaves, respectively. Conversely, a 16-h incubation was employed for the embryogenic callus of various cultivars including Sugraone, Crimson Seedless, Merlot, and Thompson Seedless, as reported by Scintilla et al. (2022) and Tricoli and Debernardi (2024). Close to our findings, Zhao et al. (2016) explored varying incubation times (6 h, 10 h, 14 h, 18 h) during enzyme digestion for protoplast isolation from *V. vinifera* cv. Rizamat leaf mesophyll tissues, identifying the optimal incubation duration as 14 h (Zhao et al. 2016).

However, our study diverges from this precedent, revealing that an 8-h digestion period proved optimal for Chardonnay grapevine cultivars. This discrepancy highlights the nuanced nature of protoplast isolation methodologies, necessitating tailored approaches to suit specific grapevine varieties and experimental objectives.

Following enzymatic digestion of leaf material, the resulting mixture contains a complex mix of components, which includes intact protoplasts (isolated plant cells), undigested material, damaged protoplasts, and cellular debris. These unwanted components can significantly impact the health and functionality of the protoplasts for downstream applications (Chen et al. 2023). The purification process aims to obtain a population of highly viable and intact protoplasts suitable for further experiments (see Factors 6 and 7). Since the initial purification step typically focuses on removing large debris like undigested cell clusters, we first implemented filtration to separate cell clumps and undigested tissues, utilizing mesh filters with varying pore sizes (40 and 70 µm). While the 40 µm filter effectively removed clumps and large cell debris, it resulted in a low yield due to larger protoplasts exceeding 40 µm. Our investigation revealed that a pore size of 70 µm was optimal for preventing the breakage of larger protoplasts, thereby maximizing protoplast yield. This finding aligns with similar pore sizes reported in other grapevine studies (Zhao et al. 2016; Tricoli and Debernardi 2024; Scintilla et al. 2022).

**Fig. 6:**
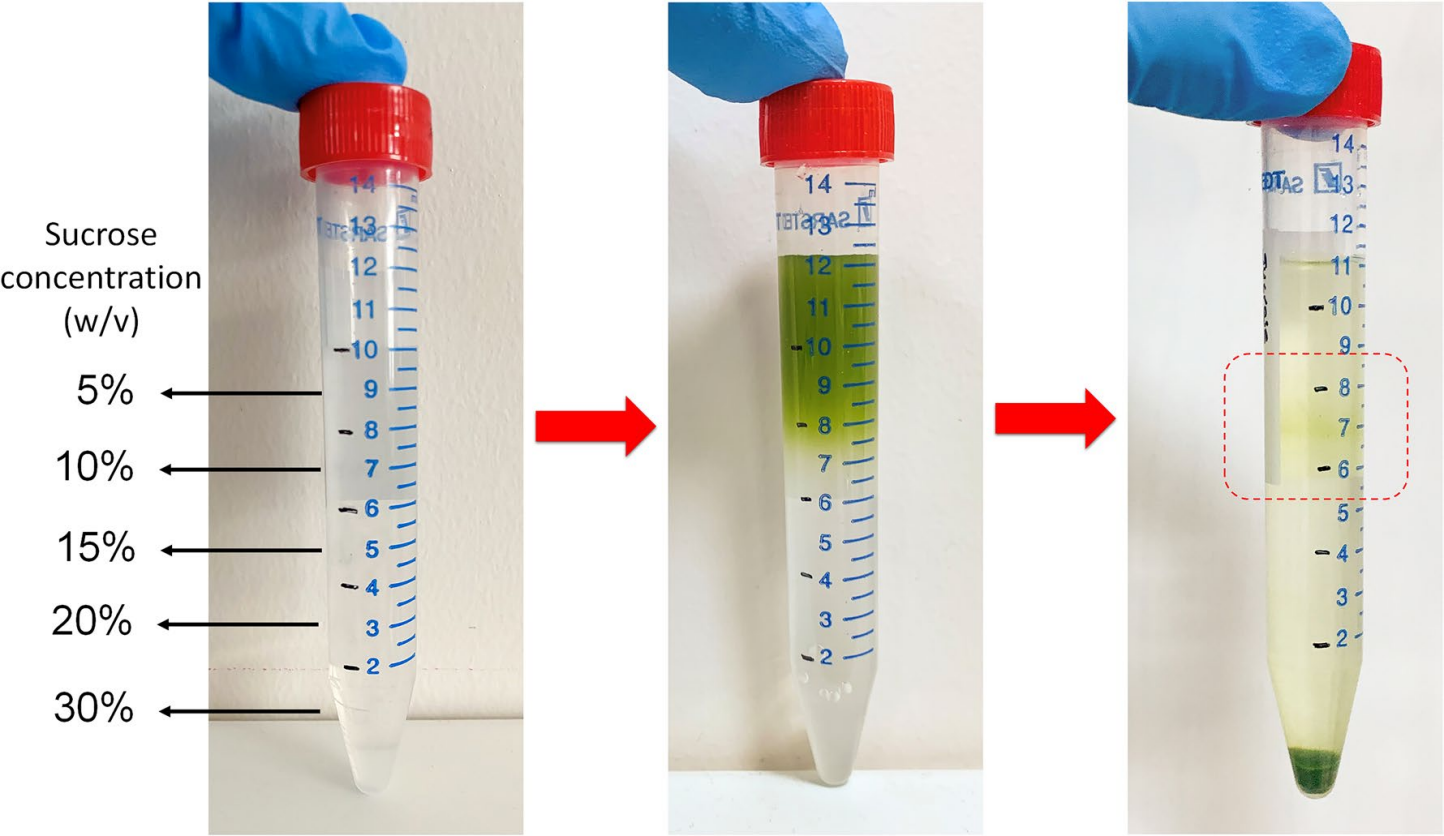
Protoplast purification by sucrose gradient centrifugation method. The red dashed box highlights the protoplast interface, which is the boundary between the protoplasts and the sucrose gradient layers

Previous studies have utilized ice incubation and sucrose gradients to separate intact protoplasts from cellular debris following digestion (Shao et al. 2023; Panda et al. 2024). In certain protocols, only the centrifugation step was employed after washing with W5 buffer (Biswas et al. 2022). Hence, three different separation methods, ice incubation, sucrose gradient, and centrifugation, were evaluated to ascertain the most effective method for protoplast purification. We incubated protoplasts on ice for 30 min to settle by gravity after resuspension in W5 buffer. This process yielded protoplasts in the supernatant and pellet fractions; microscopic observation revealed many healthy protoplasts in the supernatant alongside cell debris and broken protoplasts. Contrary to previous studies (Wu et al. 2020; Shao et al. 2023), ice incubation alone was insufficient for separating viable protoplasts from degraded cellular components. Implementing of a sucrose gradient also did not improve viable protoplast yield despite producing a visible band of protoplasts (Fig. 6) contrary to previous reports (Jeong et al. 2021; Moon et al. 2021; Panda et al. 2024). In contrast, we predominantly recovered many damaged protoplasts rather than healthy ones.

We monitored yield and viability throughout the isolation process to investigate the source of the high level of broken protoplasts and cell debris. We identified filtration as the primary culprit. Mechanical squeezing of the digestion mixture using a pipette tip, aimed at increasing protoplast yield, resulted in significant cell damage. Eliminating this step not only reduced broken protoplasts and debris but also surprisingly increased yield. Like Biswas et al. (2022), we achieved effective separation of protoplasts solely through centrifugation without additional ice incubation or sucrose gradients. Therefore, separation occurred in a shorter time following the filtration of protoplasts after digestion.

**Fig. 7:**
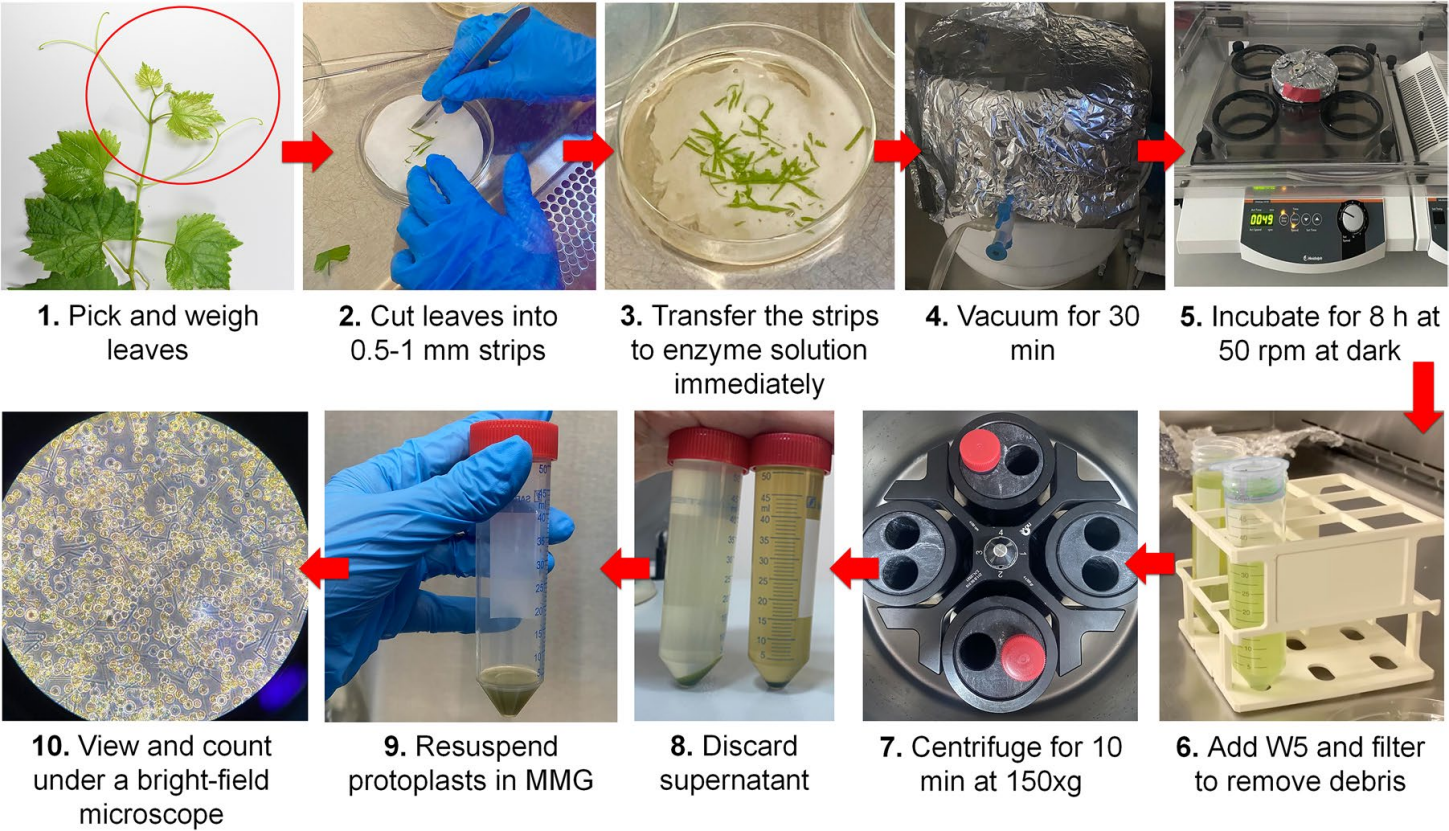
Workflow of optimized protoplast isolation protocol

Based on the optimum factors investigated, the maximum protoplast yield was recorded as 75 × 10^6^/g weight by applying the optimized protoplast isolation protocol. Figure 7 depicts a schematic representation of the protocol. Full details of the protocol can be found in Supplementary Information S1.

**Fig. 8:**
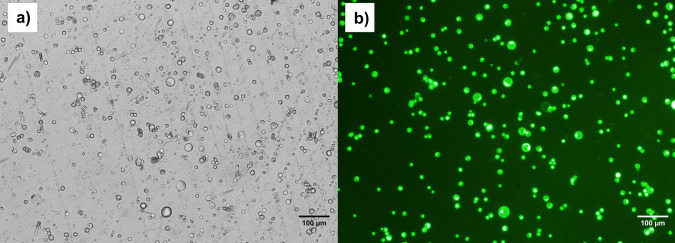
Imaging of viable protoplasts under the bright field and fluorescence microscope

Following the protoplast isolation, fluorescence microscopy analysis revealed that the protoplasts exhibited a high survival rate, with 91% ± 2.42% viability determined by FDA staining. The results demonstrated consistent viability values with minimal variation, indicating high reproducibility of the protocol under the conditions tested. This high viability indicates that the isolated protoplasts retained their integrity and functionality, fulfilling the stringent requirements of our experimental protocols. The relatively low standard deviation indicates minimal variability between replicates, suggesting that the isolation protocol is both reliable and reproducible under the conditions tested. A fluorescence microscopy image of the viable protoplasts is shown in Fig. 8. Furthermore, the viability of these same protoplasts was recorded as 78% ± 8.51% one day later, as determined by FDA staining. Previous studies have reported varying protoplast viability rates, with Zhao et al. (2016) and Malnoy et al. (2016) identifying enzyme concentration as a critical factor influencing protoplast quality. Both studies found that grapevine protoplasts had high viability, with Zhao et al. (2016) reporting 96% viability and Malnoy et al. (2016) reporting 90% viability using enzyme concentrations of 1.5% cellulase and 0.4% macerozyme. Zhao et al. (2016) further optimized enzyme concentrations, concluding that 1.5% cellulase and 0.4% macerozyme were optimal for maximizing protoplast viability. In contrast, Bertini et al. (2019) increased enzyme concentrations to 2% cellulase and 1% macerozyme, achieving a viability rate above 80%, while Najafi et al. (2023) did not report viability results for these enzyme concentrations. Interestingly, Scintilla et al. (2022) achieved viability rates exceeding 99% by reducing the enzyme concentrations to 1% cellulase and 0.3% macerozyme. These studies suggest that variations in enzyme concentrations can influence protoplast viability, likely through their effect on osmotic pressure during isolation. Notably, while these studies report initial viability rates, the viability of protoplasts one day after isolation is often unspecified, making it difficult to compare long-term viability across different conditions.

### Transformation efficiency in protoplasts

**Fig. 9:**
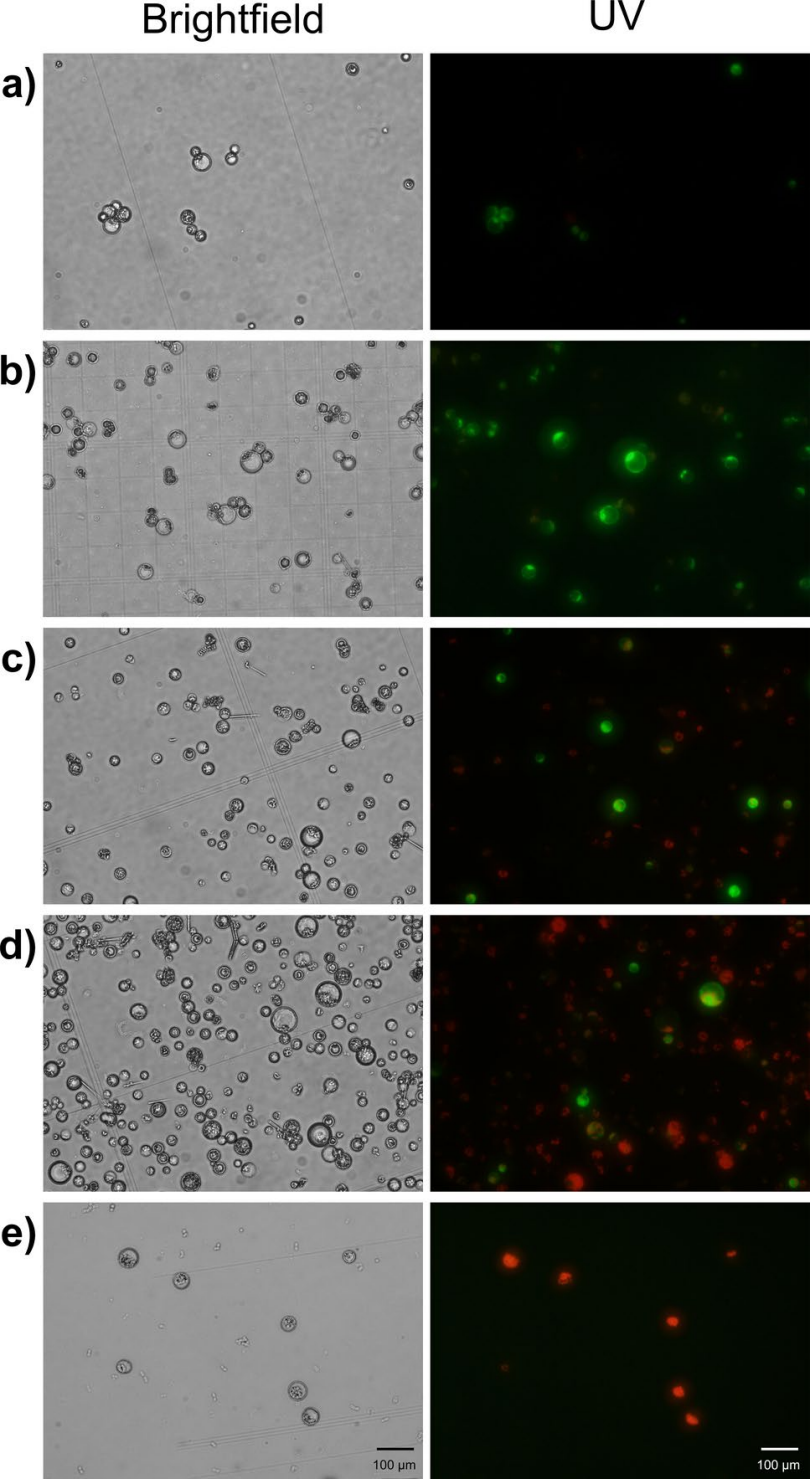
Protoplasts observed under bright field and fluorescence microscopy following transformation with different protoplast numbers. **a** 2.5 × 10^5^ protoplasts. **b** 5 × 10^5^ protoplasts. **c** 1 × 10⁶ protoplasts. **d** 2 × 10⁶ protoplasts. **e** Negative control (−): protoplasts with PEG solution only

**Fig. 10:**
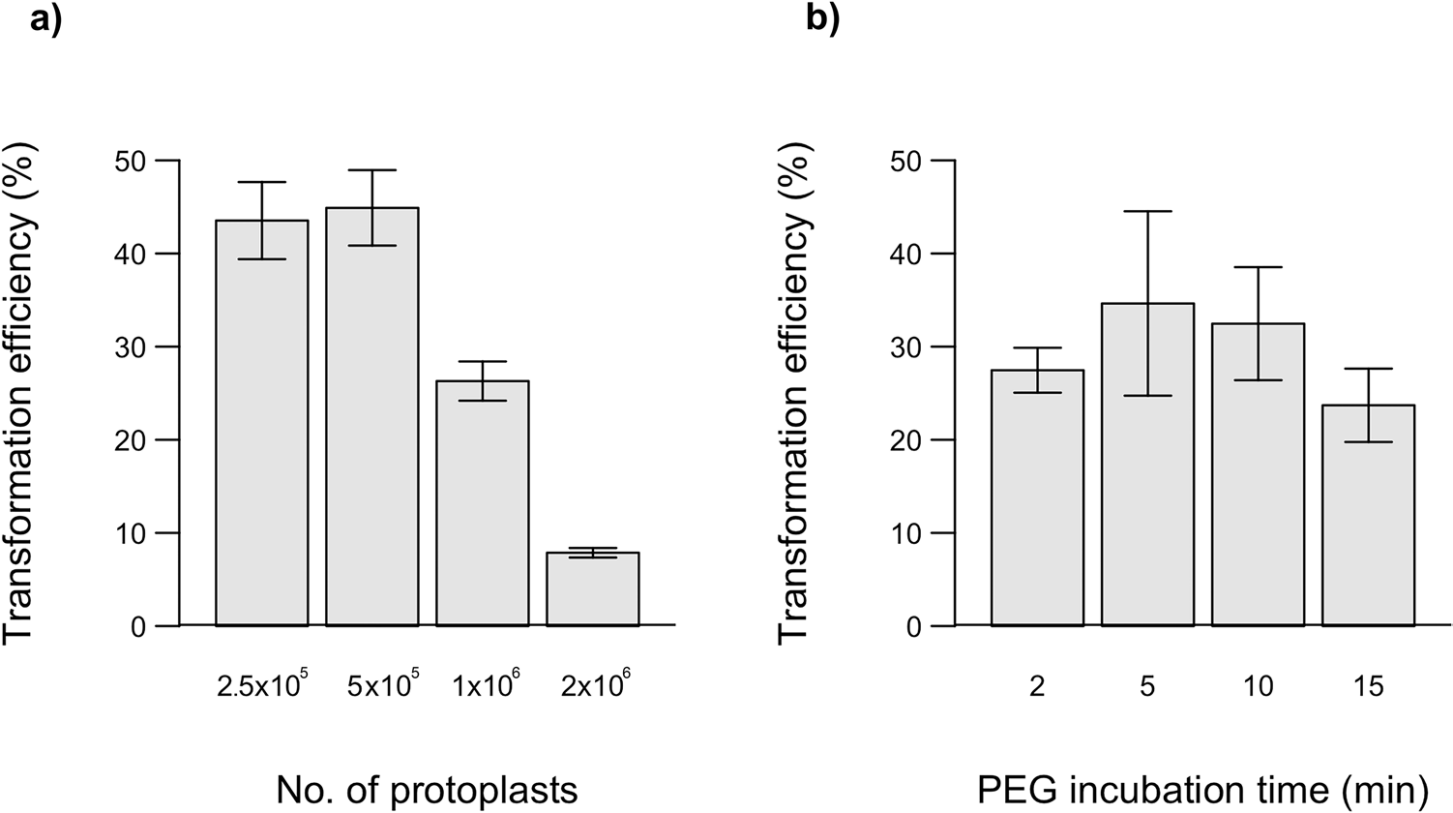
Comparison of transformation efficiency depend on **a** protoplast number and **b** PEG incubation time

An efficient transformation protocol for grapevine protoplasts was developed through a series of experiments. This protocol involved progressive optimization steps. In line with previous studies highlighting the importance of protoplast number on transformation efficiency (Zhao et al. 2016; Huo et al. 2017; Gou et al. 2020), we tested four protoplast numbers: 2.5 × 10^5^, 5 × 10^5^, 1 × 10⁶, and 2 × 10⁶ protoplasts. The highest transformation efficiency was observed with 5 × 10^5^ protoplasts. Transformed protoplasts at various numbers are shown in both bright-field and fluorescence microscopy images (Fig. 9). As illustrated in Fig. 10a, transformation efficiency decreased when the protoplast number exceeded 5 × 10^5^.

Comparing our results to those of Zhao et al. (2016), who reported a transformation efficiency of 94% using 6 × 10^4^ cells/mL in wild Chinese and Rizamat grapevine cultivars, we observed a different trend. When Zhao et al. (2016) increased the protoplast concentration to ~ 5 × 10^5^ cells/mL, their transformation efficiency dropped to around 40%. In contrast, our study achieved the highest transformation efficiency at 5 × 10^5^ protoplasts/mL. In addition to using different cultivars, Zhao et al. (2016) used 40 µg plasmid DNA, whereas we used 15 µg. These results suggest that transformation efficiency may be genotype-dependent, as well as influenced by other experimental factors such as plasmid DNA concentration and protocol variations.

Similarly, Malnoy et al. (2016), working with Chardonnay, performed transformations using 2 × 10^5^ protoplasts, which they considered optimal, although they did not report transformation efficiency. Najafi et al. (2023), in their study with Thompson Seedless protoplasts from embryogenic callus, reported a transformation efficiency of 17% using 2 × 10^5^ protoplasts. In contrast, Tricoli and Debernardi (2024) used 1 million protoplasts from embryogenic callus of various cultivars but did not report the transformation efficiency. Comparing our results with those of other grapevine studies, it is evident that effective transformation can be achieved without the need for an excessively high number of protoplasts. These findings suggest that very high protoplast concentrations may hinder transformation efficiency, potentially due to increased cell aggregation or competition for transformation reagents (Zhao et al. 2016).

Four different incubation times (2, 5, 10, and 15 min) were tested to evaluate the impact of PEG incubation time on transformation efficiency in Chardonnay protoplasts. The transformation efficiencies corresponding to each PEG incubation time are shown in Fig. 10b. Transformation efficiency increased proportionally with an incubation time of up to 5 min; however, it began to decline beyond this point.

Similarly, Zhao et al. (2016) tested various PEG incubation times ranging from 1 to 40 min in grapevine protoplasts derived from mesophyll tissues of the Chinese and Rizamat grape varieties. They found that the optimal incubation time was 5 min, which resulted in the highest transformation efficiency (over 80%). Transformation efficiency decreased when the incubation time exceeded 5 min. In contrast, Malnoy et al. (2016), who worked with Chardonnay protoplasts, suggested that the optimal PEG incubation time for transforming protoplasts derived from callus and mesophyll tissues was 20 min, though they did not provide conclusive data on transformation efficiency.

Other grapevine studies working with different varieties also indicated that the optimal PEG incubation time for protoplasts from callus tissues fell within the range of 15–20 min. However, these studies did not report transformation efficiencies (Bertini et al. 2019; Tricoli and Debernardi 2024). Based on these results, a 20-min PEG incubation time was found to yield high transformation efficiency in protoplasts derived from grapevine callus, while incubation times longer than 5 min appeared to cause damage to protoplasts from grapevine mesophyll tissues, leading to reduced efficiency. These findings highlight the variability in optimal PEG incubation times, not only between different grapevine varieties but also within the same variety, depending on the type of explant tissue used.

The incubation buffer plays a crucial role in the success of transformation. In preliminary experiments, protoplasts incubated in MMG buffer for transient gene expression did not transform; instead, they ruptured and lost shape. In contrast, when protoplasts were incubated in a W5 buffer, the transformation was successful, and the protoplasts remained viable and retained their shape, indicating better health and stability. Similarly, Zhao et al. (2016) and Malnoy et al. (2016) successfully used W5 buffer for incubation after transformation for transient gene expression.

**Fig. 11:**
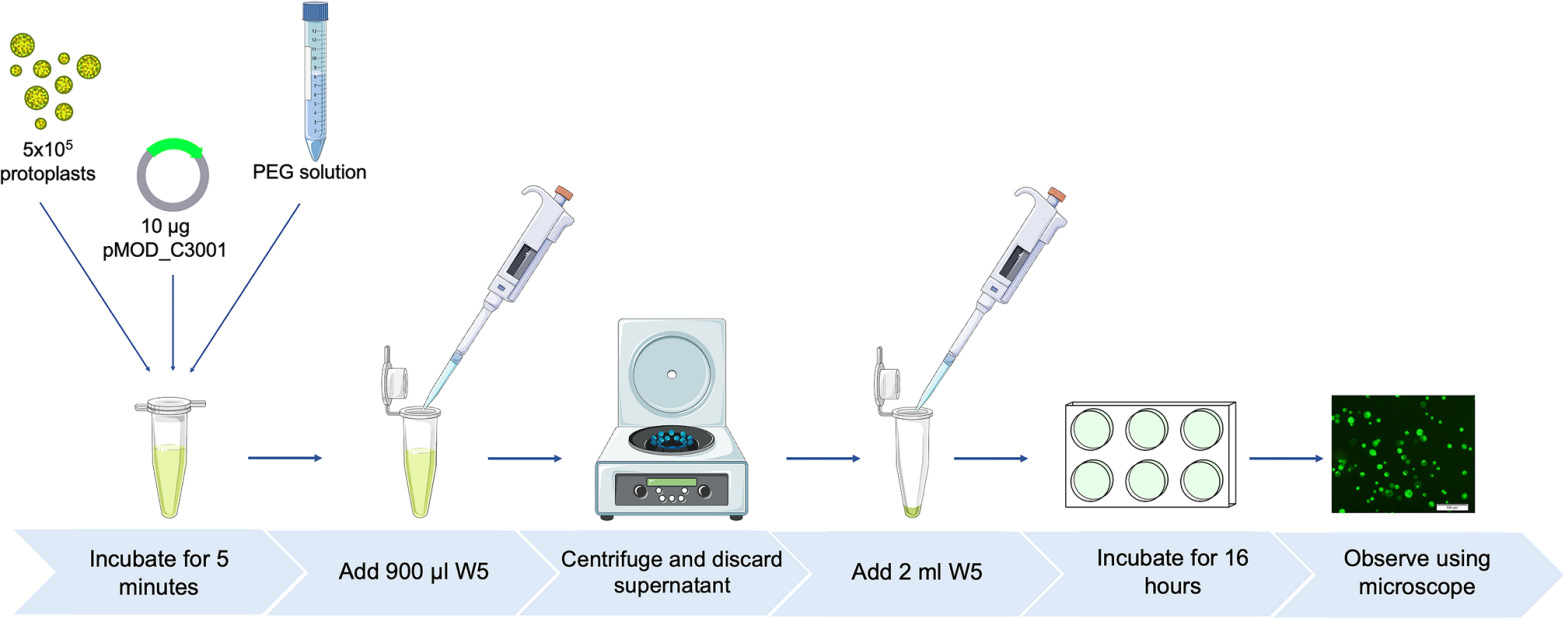
Workflow of optimized protoplast transformation protocol

To investigate the effect of incubation duration on transformation efficiency, protoplasts were incubated for 16 and 20 h to assess transient gene expression. The 16-h incubation period was identified as optimal, as protoplasts showed green fluorescence at this time point. In contrast, no significant green fluorescence was observed after 20 h of incubation. Unlike our study, Zhao et al. (2016) observed transient gene expression with GFP in protoplasts from wild Chinese and Rizamat grape varieties after 20–25 h of incubation. The absence of transient gene expression in Chardonnay protoplasts after 20 h highlights that protocol optimization can vary depending on the grape variety. The workflow of the optimized transformation protocol successfully applied to Chardonnay protoplasts is illustrated in Fig. 11. A detailed step-by-step protocol is provided in Supplementary Information S2.

### Screening for GFP gene in transformed protoplasts

**Fig. 12:**
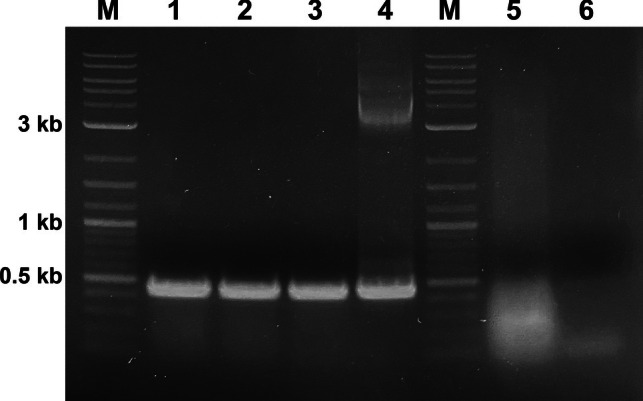
Agarose gel electrophoresis of PCR products for GFP detection in transformed grapevine protoplasts. M: 1 kb DNA ladder; 1–3: PCR products from GFP-transformed protoplasts (5 × 10.^5^ cells); 4–6: PCR products from GFP-transformed protoplasts (1 × 10⁶ cells); 7: Positive control plasmid containing GFP; 8: Negative control (wild-type Chardonnay)

In this study, GFP gene insertion was confirmed with PCR, in addition to fluorescence microscopy. The gel image shown in Fig. 12 illustrates the PCR results used to detect the presence of GFP in transformed grapevine protoplast at concentrations of 5 × 10^5^ and 1 × 10⁶ cells. Gel imaging revealed that the expected PCR product of 473 bp was successfully amplified in all DNA samples from GFP-containing protoplasts, confirming the presence of GFP. Similar PCR-based GFP verification has been reported in grapevine protoplast regeneration study (Najafi et al. 2023). However, in contrast to our approach, they isolated genomic DNA from the leaves of regenerated transformed plants. This suggests that GFP can be effectively detected not only in protoplasts but also in regenerated plants using PCR-based methods. Moreover, PCR validation of GFP has been widely applied in other plant species, such as citrus (Omar, Song, Grosser 2007) and carnation (Adedeji et al. 2022). These studies further support the versatility of PCR as a reliable method for confirming the presence of GFP in genetically transformed plants.

### Induction of microcalli from protoplasts

Protoplast regeneration involves several complex stages, including cell wall recovery, re-entry into the cell cycle, proliferation of pluripotent callus, shoot regeneration, de novo root formation, and plantlet establishment (Damm and Willmitzer 1988; Lin et al. 2018a, b; Jeong et al. 2021). Each stage requires careful optimization of the protoplast isolation protocol and culture conditions, such as growth media, hormone combinations, and environmental factors.

**Fig. 13:**
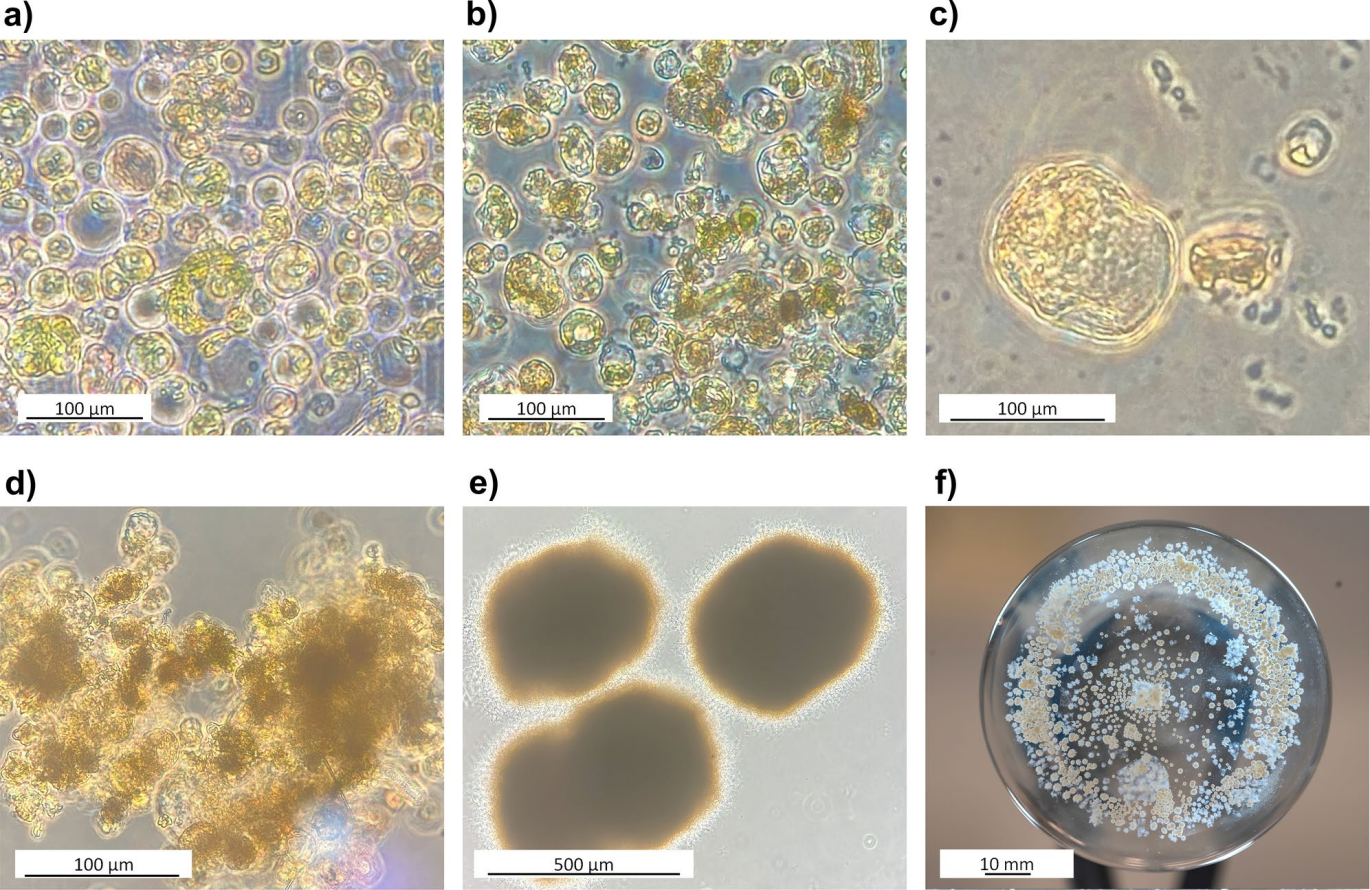
Microcalli formation. **a** Isolated protoplasts in culture medium at the initial stage. **b** After 15 days. **c** Initiation of cell division after 30 days. **d** Microcalli formation approximately 90 days later. **e** Microcalli appeared as clustered masses within 4 months. **f** Microcalli visible to the naked eye as small white structures by the end of the 120-day period

This study evaluated protoplasts derived from Chardonnay mesophyll tissues under different culture conditions for their callus induction and plant regeneration potential. Both transformed and untransformed protoplasts were cultured on solid and liquid MS media, supplemented with 2 mg/L 2,4-D and 0.5 mg/L BA to promote microcallus formation. Microcalli developed on the feeder layer, and calli were obtained when the microcalli were transferred to liquid MS media with the same hormone concentrations. However, despite the formation of microcalli, these calli did not differentiate into embryos capable of regenerating roots or shoots. The initial state of the protoplasts in the culture medium, as observed under the microscope, is shown in Fig. 13a. The protoplasts began to deteriorate within 15 days (Fig. 13b). After 15 days, protoplasts that initiated cell division continued to divide for up to 30 days (Fig. 13c), and 90 days later, microcalli appeared as clustered masses (Fig. 13d). By 120 days, microcalli formation became more pronounced (Fig. 13e) and was visible to the naked eye as small white structures in the flask (Fig. 13f). However, no root or shoot formation was observed from the calli by the end of 120 days.

To further assess the potential for callus growth, the calli were transferred to liquid ½ MS and Lloyd’s medium, containing hormone combinations of 1 mg/L BA, 0.25 mg/L NAA, and 1 mg/L TDZ. The combination of 1 mg/L TDZ and 0.25 mg/L NAA promoted callus growth more effectively than other treatments, although again, the calli did not differentiate into embryonic forms. Moreover, the Lloyd’s medium showed little to no significant effect on callus growth.

Due to their high regenerative potential in grapevine studies, somatic embryogenic calli have been identified as favorable explants for protoplast isolation (Reustle, Harst, Alleweldt 1995). Several studies have successfully regenerated grapevine plants from protoplasts isolated from somatic embryogenic calli (Bertini et al. 2019; Scintilla et al. 2022; Najafi et al. 2023; Tricoli and Debernardi 2024). It is hypothesized that the methylation state of the source tissue influences the regenerative capacity of protoplasts, with lower methylation correlating with higher regeneration potential (Osorio-Montalvo, Sáenz-Carbonell, De-la-Peña 2018; Derman and Vivier 2022). Specifically, tissues with lower differentiation levels, such as meristems, zygotic embryos, and anthers, tend to have lower DNA methylation and higher embryogenic potential. Additionally, oxidative stress has been reported to significantly affect the recalcitrance of grapevine protoplasts by impacting cell viability and regeneration potential. However, the somatic embryogenic callus formation process is both time-consuming and time-dependent, as immature inflorescences are only available for a short period (Derman and Vivier 2022). Despite these limitations, somatic embryogenesis remains the only explant type capable of complete plant regeneration from protoplasts.

Reports on grapevine regeneration from mesophyll cell-derived protoplasts are limited, and successful regeneration from leaf protoplasts has yet to be achieved (Shimizu 1985; Lee and Wetzstein 1988; Barbier and Bessis 1990; Katsirdakis and Roubelakis-Angelakis 1992). Although successful plant regeneration from mesophyll protoplasts has been reported in other species (Mukami et al. 2022), difficulties in obtaining embryo-like structures from mesophyll protoplasts are consistently noted. Similar to our study, Barbier and Bessis (1990) also isolated protoplasts from *Chardonnay* mesophyll tissue, observing progression only to the cell division stage. However, they used a different method (liquid MS medium without a feeder layer). They found that concentrations of 2,4-D below 1.5 mg/L and NAA above 3.0 mg/L were detrimental to mitotic activity.

Shimizu (1985) promoted cell wall regeneration and cell division in protoplasts derived from mesophyll tissue of the *Koshu Sanjaku* cultivar using liquid B5 medium supplemented with 1 mg/L 2,4-D and 0.5 mg/L BA. Similarly, Katsirdakis and Roubelakis-Angelakis (1992) achieved cell division in protoplasts from *Sultanina* using a liquid MS medium supplemented with BA and NAA hormones. In another study, Lee and Wetzstein (1988) achieved microcallus formation in a B5 medium using the embedded method with protoplasts isolated from *Cabernet Sauvignon* mesophyll tissue. In our study, we also observed microcallus formation using similar hormone concentrations in MS medium, suggesting that MS medium could be used as an alternative to B5 medium for protoplast culture. In general, these studies show that while the use of liquid media is sufficient for regenerating the cell wall and promoting cell division in protoplasts, the formation of microcalli requires plating protoplasts on solid media plates, often using embedding techniques. Our findings also confirm that a higher concentration of 2,4-D relative to BA is beneficial for callus formation and that the feeder layer method is effective for promoting micro callus formation.

It has also been reported that the choice of culture techniques is crucial in determining the efficiency of plant regeneration from protoplasts, particularly in recalcitrant species like grapevine. The composition of the growth medium and its form are key parameters in protoplast regeneration. In their grapevine studies, Tricoli and Debernardi (2024) and Scintilla et al. (2022) successfully regenerated whole plants from protoplasts using feeder cells. In our study, we also aimed to evaluate the effect of the feeder layer on mesophyll protoplasts. However, no root or shoot formation was observed despite using the feeder layer. This could be attributed to differences in the protoplast source rather than variations in the media used.

In conclusion, our study demonstrates the challenges of grapevine protoplast regeneration, which involves a complex interplay of genetic, physiological, and environmental factors. We have established a detailed protocol for optimal protoplast isolation. We also demonstrated the viability of protoplasts after 24 h, providing flexibility for subsequent use of these protoplasts in further experiments. Additionally, we refined transformation parameters for these protoplasts using GFP as a model gene. Finally, we successfully induced micro callus formation from *Chardonnay* mesophyll-derived protoplasts, although the lack of root or shoot formation highlights this species’ recalcitrant nature of regeneration. Future research should focus on refining hormone combinations and culture conditions to promote somatic embryogenesis, as well as investigating genetic factors that could enhance the regenerative potential of protoplasts. By addressing these challenges, we may improve the efficiency of protoplast-based regeneration systems in grapevine and other recalcitrant species.

## Supplementary Information

Below is the link to the electronic supplementary material.

Supplementary file1 (PDF 137 KB)

## Data Availability

The authors confirm that all experimental data supporting the findings of this article are available within the article.
