# Supplementary material for: Establishing a reliable protoplast system for grapevine: isolation, transformation, and callus induction
Source: Protoplasma. 2025 Apr 25;262(6):1401–17. doi: 10.1007/s00709-025-02069-7 (PMC12535509; doi:10.1007/s00709-025-02069-7)
Supplement: Supplementary file 1 — Supplementary file1 (PDF 137 KB) [file 709_2025_2069_MOESM1_ESM.pdf]

Establishing a Reliable Protoplast System for Grapevine: Isolation, Transformation, and Callus Induction

Gulsen Kolasinliler[1], Cengiz Akkale[2], Hilal Betul Kaya[1*]

[1]Department of Bioengineering, Faculty of Engineering, Manisa Celal Bayar University, Manisa, Türkiye
[2]Izmir Biomedicine and Genome Center, Türkiye

[*]Corresponding author: hilalbetul.kaya@cbu.edu.tr, +902362012461
ORCID ID: 0000-0002-2543-7212

Supplementary Information (S1)

Protoplast Isolation Protocol

1. Weigh 20 mg of freshly cut young leaves per mL of enzyme solution.

> ***Note 1:*** *Enzyme solution: 1.5% cellulase R10 (Yakult, Tokyo, Japan), 0.75% macerozyme R10 (Yakult, Tokyo, Japan), 0.6 M mannitol, 10 mM $CaCl_2$, 0.1% BSA, and 10 mM MES (pH 5.7).*

> ***Note 2:*** *1 g of leaves yields enough protoplasts for about 150 transformations.*

2. Wash the leaves thoroughly with sterile distilled water (in a biosafety cabinet) and dry them on sterile filter paper.

> ***Note 3:*** *Wash the leaves gently to avoid tissue damage.*

3. Cut the leaves into 0.5–1.0 mm strips using a razor blade on the filter paper.

> ***Note 4:*** *Remove the petiole of the leaves. Use a new, sharp razor blade for each experiment.*

4. Transfer the cut leaves immediately into the enzyme solution in a petri dish to minimize drying and browning.

5. Vacuum the leaf strips in a desiccator at approximately 400–500 mmHg for 30 minutes in the dark, with vacuum cycles alternating every 10 minutes.

> ***Note 5:*** *Slowly turning the vacuum on and off helps prevent cell breakage and allows the enzyme solution to infiltrate the cells more effectively by minimizing prolonged exposure to vacuum and facilitating gradual tissue penetration.*

6. Incubate the strips in the enzyme solution for 8 hours, in the dark at ~50 rpm on an orbital shaker.

7. Add an equal volume of W5 buffer and gently shake for 1 minute to stop digestion and facilitate the release of protoplasts.

> ***Note 6:*** *W5 buffer: 2 mM MES (pH 5.7), 154 mM NaCl, 5 mM glucose, 125 mM* $CaCl_2$*, and 5 mM KCl.*

8. Filter the protoplasts through a 70 µm nylon mesh and collect them in a Falcon tube.

> ***Note 7:*** *Pre-wet the nylon mesh with 1 mL of W5 buffer before filtration.*

9. Centrifuge the protoplasts at 150 × g for 5 minutes at room temperature (RT) in a swinging bucket rotor with gentle acceleration and deceleration.

10. Discard the supernatant into a sterile flask.

> ***Note 8:*** *Centrifuging the supernatant again increases the protoplast yield.*

11. Collect the protoplasts (the pellet) in a 50 mL conical tube and resuspend them in 2 mL of MMG buffer.

> ***Note 9:*** *MMG buffer: 4 mM MES (pH 5.7), 0.4 M mannitol, 100 mM* $CaCl_2$ *and 15 mM* $MgCl_2$

Supplementary Information (S2)

PEG-Mediated Protoplast Transformation Protocol

1. In a 2 mL round-bottom Eppendorf tube, gently mix:
   - 5×10^5 protoplasts (200 µL)
   - 10 µg plasmid DNA (30 µL)
   - 230 µL of 40% PEG 4000 solution

   Note 1: *Avoid vortexing. Use round-bottom tubes for optimal and smooth mixing; they also facilitate easier supernatant removal after centrifugation.*

   Note 2: *PEG solution: 40% (w/v) PEG-4000 in MMG buffer*

2. Incubate the mixture at room temperature for 5 minutes.

3. Add 900 µL of W5 buffer to stop the reaction and gently mix.

4. Centrifuge at 100 × g for 5 minutes at room temperature using minimal acceleration and deceleration. Discard the supernatant.

5. Resuspend the protoplast pellet in 2 mL of W5 buffer.

   Note 3: *Add buffer in two steps—first 1 mL, mix gently, then add the second 1 mL to ensure homogeneity.*

6. Transfer the protoplast suspension into a well of a 6-well tissue culture plate.

7. Incubate for 16 hours at 28°C in the dark.

   Note 4: *A 16-hour incubation is sufficient for GFP-based transformation efficiency measurements. If using a different fluorescent marker, the incubation time may need to be adjusted depending on the marker's expression time. For gene expression purposes, longer incubation times (24-48 hours) may be required to allow for adequate expression of the transgene or successful gene editing.*

8. Image transformed protoplasts using a fluorescence microscope with appropriate excitation/emission wavelengths.
